# Efficacy of a bioflavonoid-enriched herbal versus 0.12% and 0.2% chlorhexidine mouthwashes in reducing peri-implant inflammation and subgingival *Porphyromonas* *gingivalis* carriage in patients with peri-implantitis

**DOI:** 10.2340/aos.v84.44870

**Published:** 2025-12-12

**Authors:** Nujud Alamry, Danah E. Aloumi, Sahar S. Alghamdi, Afrah E. Mohammed, Shatha Subhi ALHarthi, Munerah Saleh BinShabaib, Kawther Aabed, Suha Basuhail

**Affiliations:** a Department of Preventive Dental Sciences, College of Dentistry, Princess Nourah bint Abdulrahman University, Riyadh, Saudi Arabia; b Department of Pharmaceutical Sciences, College of Pharmacy, King Saud bin Abdulaziz University for Health Sciences, Riyadh, Saudi Arabia; c King Abdullah International Medical Research Center, Riyadh, Saudi Arabia; d Pharmaceutical Care Department, King Abdulaziz Medical City, National Guard Health Affairs, Riyadh, Saudi Arabia; e Department of Biology, College of Science, Princess Nourah bint Abdulrahman University, Riyadh, Saudi Arabia; f Microbiology and Immunology Unit, Natural and Health Sciences Research Center, Princess Nourah bint Abdulrahman University, Riyadh, Saudi Arabia; g Private Dental Practice, Riyadh, Saudi Arabia

**Keywords:** Bioflavonoids, chlorhexidine, *Porphyromonas gingivalis*, peri-implantitis, probing depth

## Abstract

**Background:**

This study compared the efficacy of a bioflavonoid-enriched herbal mouthwash versus 0.12% and 0.2% chlorhexidine (CHX) in reducing peri-implant inflammation and subgingival *Porphyromonas gingivalis* (*P. gingivalis*) carriage among patients with peri-implantitis.

**Methods:**

In all, 67 individuals diagnosed with peri-implantitis were enrolled. Demographic and implant-related data were retrieved, and subgingival biofilm samples were analysed for *P. gingivalis*. All patients underwent non-surgical mechanical debridement (MD) and were then randomised into three groups: test group (bioflavonoid mouthwash, *n* = 22), control group 1 (0.12% CHX, *n* = 23), and control group 2 (0.2% CHX, *n* = 22). Participants rinsed with 15 ml of the assigned solution twice daily for 90 days. Clinical parameters, including modified plaque index (mPI), modified bleeding index (mBI), probing depth (PD), and crestal bone loss, were recorded at baseline and after 90 days. Statistical significance was set at *P* < 0.05.

**Results:**

Significant reductions in mPI, mBI, and PD were observed across all groups compared with baseline (*P* < 0.05). The follow-up scores for these parameters were significantly lower in the test group compared with both CHX groups (*P* < 0.05). At baseline, *P. gingivalis* was detected in 79.1% of participants. After 90 days, *P. gingivalis* carriage was lower in the test group (13.6%) compared with control group 1 (50.0%) and control group 2 (54.5%).

**Conclusion:**

Prescription of a bioflavonoid-enriched herbal mouthwash following MD is more effective in reducing peri-implant inflammation and subgingival *P. gingivalis* compared with 0.12% and 0.2% CHX rinses.

## Introduction

Peri-implantitis is a biofilm-induced pathological condition characterised by inflammation of the peri-implant mucosa and progressive loss of supporting bone around osseointegrated implants, ultimately jeopardising their long-term survival [1, 2]. Despite high success rates of dental implants, peri-implant diseases (peri-implant mucositis and peri-implantitis) remain a major clinical challenge, with peri-implantitis prevalence ranging between 18–20% of patients and approximately 9–12% of implants after 5–10 years in function [3]. The aetiology of peri-implantitis is multifactorial; however, the role of poor oral hygiene maintenance and microbial biofilms dominated by Gram-negative anaerobic pathogens such as *Porphyromonas gingivalis* (*P. gingivalis*) has been firmly established [4–8]. It is also worth mentioning that *P. gingivalis* is a keystone pathogen in the etiopathogenesis of periodontitis [9]; and is also central in peri-implant dysbiosis [10, 11].

The clinical management of peri-implantitis primarily aims to reduce inflammation, arrest further crestal bone loss (CBL), and preserve implant function. Non-surgical mechanical debridement (MD) remains the cornerstone of treatment, employing handheld instruments such as plastic curettes [12–14]; however, a consensus on whether surgical MD is superior to non-surgical MD for the management of peri-implantitis is yet to be reached [15]. However, removal of the oral biofilm from implant surfaces is challenging due to their micro-structured morphology and the intricate design of prosthetic reconstructions, often resulting in incomplete debridement [16]. Consequently, adjunctive antimicrobial therapies following non-surgical MD have been widely investigated for the management of periodontal and peri-implant inflammatory conditions such as periodontitis and peri-implantitis, respectively [17–20]. Chlorhexidine (CHX) is regarded as the gold standard chemical plaque control agent because of its broad-spectrum antibacterial activity, substantivity, and proven clinical efficacy [17–19]. Traditionally, 0.12% and 0.2% CHX mouth rinses are the most frequently used concentrations in clinical practice, with the latter believed to offer stronger antimicrobial effects [21]. According to Esposito et al. [22], CHX, when used adjunctively, can modestly reduce peri-implant mucosal inflammation and bacterial load. Recently, interest has emerged in enhancing the antibacterial and anti-inflammatory potential of natural compounds with complementary pharmacological actions [23]. Bioflavonoids, a diverse group of plant-derived polyphenolic compounds, are known for their antimicrobial, antioxidant, and anti-inflammatory properties [24, 25]. Results from experimental studies [23, 25] have shown that bioflavonoids exert direct inhibitory effects on *P. gingivalis*, attenuate biofilm formation, and modulate host immune responses by downregulating proinflammatory cytokines. While the antibacterial and clinical efficacy of 0.12% and 0.2% CHX has been investigated in patients with periodontitis [26–29]; direct comparisons of their clinical efficacy, particularly against a bioflavonoid-enriched herbal formulation in patients with peri-implantitis remains unexplored.

The present study aimed to compare the efficacy of 0.12% CHX, 0.2% CHX, and a bioflavonoid-enriched mouthwash after MD in reducing peri-implant inflammation and subgingival *P. gingivalis* carriage in patients with peri-implantitis. It is hypothesised that in patients with peri-implantitis, rinsing with a bioflavonoid-enriched herbal mouthwash following MD is more effective in reducing peri-implant inflammation and subgingival *P. gingivalis* carriage compared [30] to rinsing with 0.12% and 0.2% CHX.

## Materials and methods

### Ethical approval

The present study was performed in accordance with the ethical principles outlined in the Declaration of Helsinki as revised in 2013 [31]. Ethical approval for the research protocol was obtained from the Ethics Board at the Princess Nourah bint Abdulrahman University, Riyadh, Saudi Arabia (Approval # HAP-01-R-059). All eligible participants were informed in detail about the purpose, procedures, potential risks, and anticipated benefits of the study. All individuals were allowed to ask questions and written informed consent was obtained from all participants before enrolment. Each participant was assured of the voluntary nature of their participation, with the right to withdraw from the study at any stage without any consequences to their standard care. Confidentiality of personal data was strictly maintained, and all information was anonymised before analysis.

### Study location and participants

The study was performed at the Department of Preventive Dental Sciences, College of Dentistry, Princess Nourah University, Riyadh, Saudi Arabia, between April 2024 and March 2025. Patients diagnosed with peri-implantitis were included in the present study.

### Inclusion and exclusion criteria

The inclusion criteria were as follows: (1) individuals aged ≥ 18 years old and (2) patients diagnosed with peri-implantitis. The diagnosis of peri-implantitis was established on clinical and radiographic criteria described elsewhere [30]. Diagnosis of peri-implantitis was based on the following criteria: peri-implant probing depth (PD) and CBL of ≥ 6 mm and ≥ 3 mm, respectively [30]. Self-reported tobacco smokers, individuals using electronic nicotine delivery systems, alcohol users, and smokeless tobacco product users were excluded. Individuals with self-reported systemic diseases, including diabetes mellitus (DM), osteoporosis, rheumatoid arthritis, cardiovascular, renal and hepatic diseases, and patients with HIV/AIDS were considered ineligible for inclusion. Individuals who reported having undergone surgical/non-surgical periodontal or peri-implant treatment, and individuals who had used antibiotics, probiotics, steroids, and non-steroidal anti-inflammatory drugs and/or bisphosphonates within the past 90 days were excluded. Moreover, patients with existing or a history of periodontitis and nursing/pregnant females were not sought.

### Demographic information and evaluation of digital dental records

A questionnaire was used to gather information on patients’ age and gender. The principal investigator evaluated digital healthcare records to collect the following information: (1) total number of implants; (2) jaw location of implant/s; (3) duration of implant/s in function; (4) depth of insertion (crestal or sub-crestal); (5) mode of prosthesis retention (cement or screw retention).

**Table 1:** Characteristics of the study cohort.

Parameters	Individuals diagnosed with peri-implantitis			
	All individuals	Test-group	Control group 1	Control group 2*
Participants (*n*)	67 individuals	22 individuals	23 individuals	22 individuals
Male:Female	40:27	15:7	13:10	12:10
Age in years	58.9 ± 9.4	55.2 ± 5.6	54.4 ± 4.1	60.5 ± 5.7
Number of implants	67 implants	22 implants	23 implants	22 implants

Posterior maxilla: implants replacing missing maxillary first or second molar.

Posterior mandible: implants replacing missing mandibular first or second molar.

**Table 2:** Peri-implant parameters at baseline and at 90 days of follow-up.

Peri-implant parameters	Baseline				90 days of follow-up			
	All individuals	Test-group	Control group 1	Control group 2	All individuals	Test-group	Control group 1	Control group 2
mPI	0.82 ± 0.07^*^	0.84 ± 0.04^§^	0.8 ± 0.03	0.8 ± 0.05	0.44 ± 0.02	0.3 ± 0.004^#^	0.5 ± 0.003	0.52 ± 0.008
mBI	0.83 ± 0.03^†^	0.82 ± 0.03^‖^	0.85 ± 0.06	0.8 ± 0.04	0.42 ± 0.06	0.21 ± 0.002^**^	0.5 ± 0.004	0.47 ± 0.006
PD (in mm)	6.4 ± 0.7 mm^‡^	6.7 ± 0.5 mm^¶^	6.2 ± 0.5 mm	6.3 ± 0.2 mm	2.5 ± 0.2 mm	1.6 ± 0.005 mm^††^	3.3 ± 0.2 mm	2.9 ± 0.1 mm
CBL (mesial)	4.5 ± 0.5 mm	4.6 ± 0.3 mm	4.3 ± 0.06 mm	4.7 ± 0.2 mm	4.47 ± 0.3 mm	4.58 ± 0.3 mm	4.32 ± 0.07 mm	4.66 ± 0.05 mm
CBL (distal)	4.52 ± 0.7 mm	4.65 ± 0.4 mm	4.4 ± 0.05 mm	4.73 ± 0.3 mm	4.6 ± 0.4 mm	4.7 ± 0.1 mm	4.4 ± 0.2 mm	4.55 ± 0.2 mm

mPI: modified plaque index; mBI: modified bleeding index; PD: probing depth; CBL: crestal bone loss.

* Compared to all individuals at 90 days of follow-up (*P* < 0.05).

† Compared to all individuals at 90 days of follow-up (*P* < 0.05).

‡ Compared to all individuals at 90 days of follow-up (*P* < 0.05).

§ Compared to the test-group at 90 days of follow-up (*P* < 0.05).

‖ Compared to the test-group at 90 days of follow-up (*P* < 0.05).

¶ Compared to the test-group at 90 days of follow-up (*P* < 0.05).

# Compared with control-group 1 (*P* < 0.05) and 2 (*P* < 0.05) at 90 days of follow-up.

** Compared with control-group 1 (*P* < 0.05) and 2 (*P* < 0.05) at 90 days of follow-up.

†† Compared with control-group 1 (*P* < 0.05) and 2 (*P* < 0.05) at 90 days of follow-up.

### Grouping and randomisation

Patients diagnosed with peri-implantitis were randomly allocated into three parallel groups based on the type of postoperative oral rinse prescribed following non-surgical MD. Randomisation was performed using a computer-generated random sequence (IBM SPSS Statistics for Windows, Version 27.0; IBM Corp., Armonk, NY, USA). Allocation concealment was ensured by placing group assignments in sequentially numbered opaque, sealed envelopes that were opened only after debridement was completed. In the test group, participants were prescribed a non-alcoholic, bioflavonoid-enriched mouthwash (CURAPROX PERIOPLUS+ ZERO, Curaden International AG, Kriens, Switzerland; distributed in Northampton, United Kingdom). Patients were instructed to rinse with 15 mL of the solution for 60 s twice daily (every 12 h) over 90 days. In control group 1, participants were prescribed 0.12% CHX mouthwash (Peridex®, 3M ESPE, St. Paul, Minnesota, USA) with identical rinsing instructions (15 mL, twice daily for 90 days). In control group 2, participants were prescribed 0.2% CHX mouthwash (Corsodyl®, GlaxoSmithKline Consumer Healthcare, Brentford, Middlesex, United Kingdom), again with the same rinsing regimen. Participants in all groups were advised not to eat, drink, or rinse with water for at least 30 min after using the assigned mouthwash. Brushing and flossing techniques were explained and provided in written format to all participants after MD.

### Collection of peri-implant subgingival biofilm samples and identification of Porphyromonas gingivalis

Subgingival biofilm (SB) samples were obtained from the peri-implant sulcus of all implants diagnosed with peri-implantitis. Prior to sampling, supragingival plaque was carefully removed with sterile cotton pellets, and the area was isolated with cotton rolls to prevent saliva contamination. The peri-implant sulcus was gently air-dried. Sterile paper points (#30, Dentsply Maillefer, Ballaigues, Switzerland) were then inserted into the deepest peri-implant pocket and left in place for 30 s to allow absorption of subgingival plaque. For each site, two paper points were collected and immediately transferred into sterile Eppendorf tubes containing 200 µL of TE buffer (10 mM Tris-HCl, 1 mM EDTA, pH 8.0) and stored at −80°C until further processing [32]. The presence of *P. gingivalis* was determined by species-specific Polymerase chain reaction (PCR) amplification of the 16S rRNA gene. Polymerase chain reaction assays were performed using primers specific for *P. gingivalis*: Forward: 5′-AGGCAGCTTGCCATACTGCG-3′ and Reverse: 5′-ACTGTTAGCAACTACCGATGT-3′ [33]. The microbiological analyses were performed by a trained and blinded microbiologist.

### Mechanical debridement protocol

A trained investigator performed non-surgical MD of peri-implant sulci and implant surfaces. This procedure was performed after collection and SB samples, and clinical and radiographic assessment. In summary, local anaesthesia was administered using 2% lidocaine. Visible plaque and calculus and submucosal instrumentation were done with sterile plastic curettes (UNC 15, HuFriedy, Chicago, IL, USA). Where threads were accessible, short overlapping strokes were applied from apical to coronal directions.

### Assessment of peri-implant clinical and radiographic parameters

Peri-implant clinical (modified plaque index [mPI], modified bleeding index [mBI], and PD) and radiographic (CBL) parameters were assessed at baseline and after 90 days of non-surgical MD. The mPI and mBI were measured using the protocol described by Mombelli et al. [34]. The mPI was scored as follows: 0 = No detection of plaque; 1 = Plaque only recognised by running a probe across the smooth marginal surface of the implant; 2 = Plaque visible to the naked eye; and 3 = Abundance of soft matter clearly visible. The mBI was scored using the following criteria: 0 = No bleeding; 1 = Isolated bleeding spot visible; 2 = Blood forms a confluent red line on the free gingival margin; and 3 = Heavy or profuse bleeding. The mBI and mPI were measured on four sites per implant (mesial, palatal/lingual, distal and buccal/facial). The PD was recorded to the nearest millimetre (mm), with peri-implantitis defined as the presence of a PD ≥ 6 mm at one or more sites in conjunction with bleeding on probing and/or suppuration. The CBL was measured on bitewing radiographs as the linear distance from 2 mm below the implant abutment junction to the crestal bone [35, 36]. All radiographs were taken using the long-cone paralleling technique [37]. A trained, blinded and calibrated investigator performed clinical (Kappa 0.83) and radiographic (Kappa 0.85) investigations.

### Sample size estimation and statistical analysis

The sample size estimation (SSE) was performed in G*Power (version 3.1.9.x; Heinrich-Heine-Universität Düsseldorf, Düsseldorf, Germany). The primary endpoint was pre-specified as the change in peri-implant PD from baseline to 90 days. Since group differences were to be evaluated across three arms, the study was powered for an omnibus one-way analysis of variance (ANOVA) on the 90-day outcome adjusted for baseline (i.e. analysis of covariance [ANCOVA] framework). For the ANOVA, a two-sided α = 0.05 and power (1−β) = 0.80 was specified. The expected standardised effect size (Cohen’s *f*) was set to *f* = 0.30. Under these assumptions, the required total sample size was approximately 63 participants (~21 individuals per group). Statistical analyses were performed using IBM SPSS Statistics for Windows, Version 27.0 (IBM Corp., Armonk, NY, USA). Two-tailed tests were used throughout with α = 0.05. Continuous outcomes were displayed as means ± standard deviations. Normality was assessed using the Shapiro–Wilk test. Group comparability was evaluated with one-way ANOVA and Bonferroni post hoc adjustment tests. P-values below 0.05 were considered statistically significant. Correlation between demographics, duration of implants in function, implant jaw location, and peri-implant clinical and radiographic parameters was assessed using linear regression analysis. A blinded statistician performed all statistical analyses.

## Results

### Patient demographics

A total of 67 individuals diagnosed with peri-implantitis were included in the present study — 22, 23, and 22 individuals with peri-implantitis were assessed in the test-group, control-group 1, and control-group 2, respectively. There was no significant difference in age among individuals in all groups. In the study cohort, 59.7% (*n* = 40) of the individuals were males.

### Implant characteristics

A total of 67 implants with peri-implantitis were assessed. All implants (*n* = 67) were platform-switched and were placed at the bone level using an insertion torque ranging between 30 and 35 Ncm. All implants had moderately rough surfaces, and had been placed by experienced clinicians. The diameter and length of all implants ranged between 4.5 and 5.2 mm and 10 and 13 mm, respectively. All implants were placed in the regions of missing first or second molars in both jaws. In the test-group, 20 and 2 implants were placed in the maxilla and mandible, respectively; and in the control-group 1, 18, and 5 implants were placed in the maxilla and mandible, respectively. In control-group 2, 19, and 3 implants were placed in the maxilla and mandible, respectively. Further, 22, 23, and 22 implants with peri-implantitis were assessed in the test-group, control-group 1, and control-group 2, respectively. In the test-group, control-group, 1 and control-group 2, the implants were placed and loaded 6.4 ± 2.3, 7.4 ± 2.8, and 6.6 ± 3.1 years ago, respectively. All implants (*n* = 67) were restored with screw-retained restorations.

### Comparison of peri-implant inflammatory parameters at baseline and 90 days of follow-up

Compared with baseline, there was a statistically significant reduction in mPI (*P* < 0.05), mBI (*P* < 0.05), and PD (*P* < 0.05) in all individuals at follow-up. At follow-up, scores of mPI (*P* < 0.05), mBI (*P* < 0.05), and PD (*P* < 0.05) were significantly higher among patients in control-groups 1 and 2 compared with individuals in the test-group. At follow-up, there was no difference in mPI, mBI, and PD among patients in control-groups 1 and 2. There was no significant difference in CBL in all individuals through the study duration.

### Identification of Porphyromonas gingivalis in the subgingival biofilm

At baseline, *P. gingivalis* was identified in SB samples from 53 (79.1%) of the patients with peri-implantitis (*n* = 67). In the test-group and control groups 1 and 2, *P. gingivalis* was identified in 16 (72.7%), 18 (78.3%), and 19 (86.4%) individuals, respectively. At follow-up, *P. gingivalis* was more often identified in SB samples from individuals in control groups 1 (*n* = 11; 50%) and 2 (12; 54.5%) compared with individuals in the test group (*n* = 3; 13.6%).

### Linear regression analysis

There was no significant correlation between patient demographics, duration of implants in function, implant jaw location and peri-implant clinical and radiographic parameters at both time intervals.

## Discussion

In summary, the present study demonstrated that adjunctive use of a bioflavonoid-enriched mouthwash following non-surgical MD resulted in greater improvements in peri-implant inflammatory parameters and more substantial reduction in subgingival *P. gingivalis* carriage compared to conventional 0.12% and 0.2% CHX rinses. All groups exhibited significant reductions in mPI, mBI, and PD at 90 days of follow-up compared with baseline; however, the magnitude of improvement was significantly greater in the test group, suggesting that the bioflavonoid formulation exerted adjunctive anti-inflammatory and antibacterial effects beyond those attributable to mechanical biofilm disruption. The significant reductions in mPI, mBI, and PD observed in the bioflavonoid group are biologically plausible given that flavonoids possess antimicrobial, antioxidant, and host-modulatory properties. Experimental studies [38–41] have confirmed that bioflavonoids directly inhibit *P. gingivalis* growth, attenuate biofilm formation, and suppress proinflammatory cytokine release. It has also been reported that flavonoids such as quercetin, naringenin, and catechins directly inhibit the growth of *P. gingivalis* through disruption of bacterial cell membranes and interference with proteolytic enzyme activity [41]. Hooper et al. [23] demonstrated that Citrox (a citrus-derived bioflavonoid complex) exhibits potent antimicrobial activity against *P. gingivalis* and other oral pathogens by altering bacterial adhesion and metabolic activity. The authors of the present clinicoradiographic study support the experimental results reported by Hooper et al. [23]. Additionally, bioflavonoids interfere with quorum sensing and extracellular polysaccharide synthesis, resulting in impaired biofilm maturation and reduced structural resilience of microbial communities on implant surfaces [42]. Moreover, results by Baykulova et al. [25] reported synergistic effects of combining bioflavonoids with CHX, resulting in enhanced suppression of *P. gingivalis* biofilm formation. However, the present study investigated a bioflavonoid-enriched mouthwash as a standalone intervention rather than in synergistic combination with CHX. Nevertheless, the possibility of additive or synergistic benefits when bioflavonoids are combined with CHX remains a promising avenue for future research. Randomised controlled trials (RCTs) with multiple arms directly comparing standalone bioflavonoids, standalone CHX, and their combined formulations would help clarify whether synergistic interactions can produce superior long-term outcomes.

The CHX is widely regarded as a safe and effective antiseptic in mouthwash formulations; however, hypersensitivity reactions, both immediate (Type I) and delayed (Type IV), have been increasingly reported [43]. The true prevalence of CHX allergy remains uncertain due to underreporting and inconsistent labelling of products, but contact sensitisation rates estimated via patch testing range from approximately 0.5 % to 1.0%, while anaphylaxis comprises a small yet notable portion of perioperative allergic events [43]. Symptoms of CHX allergy include mucosal erythema, swelling, and burning sensation [44, 45]. Although no signs or symptoms indicative of CHX allergy were observed among participants in the present investigation and formal assessment of CHX hypersensitivity was beyond the scope of this study; it is hypothesised that prescribing a bioflavonoid-enriched or herbal mouthwash represents a suitable alternative for individuals with documented or suspected CHX allergy. The authors applaud results of a recent RCT [17], which concluded that herbal oral rinses are a suitable substitute for patients with self-reported CHX allergy.

According to the present results, there was no significant difference in CBL between baseline and follow-up. This could be attributed to the short-term follow-up of the present investigation. It is hypothesised that compliance towards oral hygiene maintenance could result in a rise in peri-implant crestal bone levels following treatment of peri-implantitis; however further long-term observational studies are needed in this regard. Although non-surgical MD continues to remain the ‘gold standard’ for the management of peri-implant diseases [15]; studies [46, 47] have shown that adjuvant treatments such as photobiomodulation and photodynamic therapy (PDT) are more effective in the treatment of peri-implant mucositis and peri-implantitis than MD alone. It is hypothesised that prescription of a bioflavonoid-based oral rinse after MD with adjuvant photobiomodulation or PDT is more effective in the management of peri-implant diseases (including peri-implant mucositis) than prescribing a bioflavonoid-based oral rinse after MD alone. Stringent eligibility criteria were imposed in the present study such as exclusion of nicotinic product users, immunosuppressed individuals. It is well known that systemic conditions such as persistent hyperglycaemia (a classical sign of patients with poorly-controlled DM) and habitual use of non-combustible and combustible nicotinic products are risk factors for periodontal and peri-implant diseases [48, 49]. It is therefore likely that the outcomes of MD and postoperative use of oral rinses (herbal-based or CHX) are compromised in such populations. Further well-designed and power adjusted clinical trials are needed to assess the efficacy of herbal oral rinses in managing periodontal and peri-implant inflammatory conditions among smokers and immunocompromised patients.

## Conclusion

Prescription of a bioflavonoid-enriched herbal mouthwash following MD is more effective in reducing peri-implant inflammation and subgingival *P. gingivalis* compared with 0.12% and 0.2% CHX rinses.
